# Intersectoral policy for severe and persistent mental illness: review of approaches in a sample of high-income countries

**DOI:** 10.1017/gmh.2015.16

**Published:** 2015-08-24

**Authors:** S. Diminic, G. Carstensen, M. G. Harris, N. Reavley, J. Pirkis, C. Meurk, I. Wong, B. Bassilios, H. A. Whiteford

**Affiliations:** 1 Queensland Centre for Mental Health Research, Brisbane, Australia; 2 School of Public Health, The University of Queensland, Brisbane, Australia; 3 Melbourne School of Population and Global Health, University of Melbourne, Melbourne, Australia

**Keywords:** Intersectoral, mental health policy, mental health services, severe and persistent mental illness, whole of government

## Abstract

**Background:**

It is increasingly recognised that intersectoral linkages between mental health and other health and support sectors are essential for providing effective care for individuals with severe and persistent mental illness. The extent to which intersectoral collaboration and approaches to achieve it are detailed in mental health policy has not yet been systematically examined.

**Methods:**

Thirty-eight mental health policy documents from 22 jurisdictions in Australia, New Zealand, the United Kingdom, Ireland and Canada were identified via a web search. Information was extracted and synthesised on: the extent to which intersectoral collaboration was an objective or guiding principle of policy; the sectors acknowledged as targets for collaboration; and the characteristics of detailed intersectoral collaboration efforts.

**Results:**

Recurring themes in objectives/guiding principles included a whole of government approach, coordination and integration of services, and increased social and economic participation. All jurisdictions acknowledged the importance of intersectoral collaboration, particularly with employment, education, housing, community, criminal justice, drug and alcohol, physical health, Indigenous, disability, emergency and aged care services. However, the level of detail provided varied widely. Where detailed strategies were described, the most common linkage mechanisms were joint service planning through intersectoral coordinating committees or liaison workers, interagency agreements, staff training and joint service provision.

**Conclusions:**

Sectors and mechanisms identified for collaboration were largely consistent across jurisdictions. Little information was provided about strategies for accountability, resourcing, monitoring and evaluation of intersectoral collaboration initiatives, highlighting an area for further improvement. Examples of collaboration detailed in the policies provide a useful resource for other countries.

## Introduction

Mental disorders are the leading cause of non-fatal disease burden globally. They accounted for 175 million years lived with disability in 2010 (Whiteford *et al*. 2013) and are forecast to cost US$16 trillion in lost productivity between 2010 and 2030 (Bloom *et al*. 2011). Individuals with mental disorders experience varying severities of illness, defined by a combination of diagnosis, intensity of symptoms, duration and degree of disability experienced (National Advisory Mental Health Council, 1993). Severe mental disorders, such as psychotic and severe mood disorders, have the greatest impact on the individual, their support networks and the broader community. Severe mental disorders have been estimated to affect 4.3% of people in high-income countries and 3.0% in low- and middle-income countries (Levinson *et al*. 2010).

Severe mental disorders may be episodic or persistent. Individuals with severe and persistent mental illness (SPMI) tend to experience impaired daily functioning and an inability to cope well with the normal demands of life (Whiteford *et al*. 2014). They have high rates of substance abuse, physical health problems and premature mortality (World Health Organization, 2013; Chesney *et al*. 2014), as well as unemployment, poverty, homelessness, discrimination, social isolation and incarceration (OECD, 2014). Effective community-based care for these individuals therefore requires a coordinated range of services, including support to improve psychosocial functioning and quality of life (Liberman & Kopelowicz, 2005), and access to physical health care, housing, income security and vocational training or employment services.

In many countries, major reforms to mental health services have occurred over the past 50 years; notably, a dramatic shift in the organisation and delivery of mental health services, from institutional care to community-based rehabilitation (Adams *et al*. 2009). Formerly, many individuals with SPMI received mental health treatment, other health care, housing, vocational and social rehabilitation within one institutional setting (Whiteford, 1994). Following deinstitutionalisation, in most countries, responsibility for funding and delivering the array of health and social services required has been distributed across multiple portfolios and levels of government, and across government and non-government providers (Segal *et al*. 2010; Whiteford *et al*., 2014). This has led to fragmented service systems and poor social and functional outcomes for this population (Rosenheck *et al*., 2003; Castle, 2011).

Consequently, there is increasing recognition of the need for improved intersectoral collaboration to adequately address the support requirements and social disadvantages experienced by individuals with SPMI (Skeen *et al*., 2010; Solar & Irwin, 2010). The World Health Organization (WHO) defines intersectoral action for health as ‘a recognised relationship between part or parts of the health sector with part or parts of another sector which has been formed to take action on an issue to achieve health outcomes in a way that is more effective, efficient or sustainable than could be achieved by the health sector acting alone’ (World Health Organization, 1997, p. 3). There is growing evidence supporting the effectiveness of intersectoral collaboration in improving health and psychosocial outcomes. For example, Fuller *et al*. (2011) found that linkages between mental health and primary health care implementing direct collaborative activities, communication systems and agreed guidelines were associated with better mental and physical health and economic outcomes. Collaborative Individual Placement and Support models, where employment specialists are integrated into and co-located with mental health treatment teams, have been shown to improve rates of competitive employment for people with severe mental illness (Bond *et al*. 2008). Further, a recent systematic review of linkages between mental health and non-health sectors found that formal mechanisms generally led to positive system- and individual-level outcomes, with facilitators including improved communication between services, strong leadership, mechanisms for conflict resolution, mutual understanding, presence of a strategic plan or coordinating body, co-location of services, clear accountability and ongoing monitoring (Whiteford *et al*. 2014). Despite this evidence, and repeated calls for greater intersectoral collaboration in mental health (Whiteford, 1994; World Health Organization, 1997), it is unclear to what extent this has translated into action.

A key step in improving intersectoral collaboration between mental health and other sectors is to provide an overarching policy direction supporting this action. Mental health policies and plans are an important tool used by governments to guide delivery and reform of services (World Health Organization, 2004), with most countries having a formally endorsed policy or plan (World Health Organization, 2011). Some countries appear to have recognised the importance of intersectoral collaboration in their mental health policy documents (Adams *et al*. 2009). However, the extent to which intersectoral collaboration and approaches to achieve it are detailed in these policies has not been systematically examined.

This study aimed to systematically review approaches to intersectoral policy for people with SPMI recorded in current mental health policy documents. The study formed part of a broader project comparing mental health policy documents in Australia and countries with similar health systems, i.e. high-income, English-speaking countries with universal health systems. However, findings are considered in terms of their relevance globally, including to low- and middle-income countries. The specific aims were to:

1. Examine the extent to which intersectoral collaboration was an objective or guiding principle of mental health policy documents;
2. Identify sectors outside of mental health which are commonly acknowledged as targets for collaboration; and
3. Profile examples of intersectoral collaboration detailed in mental health policy documents.

## Method

### Defining and identifying policy documents

WHO distinguishes between mental health policies – long-ranging, visionary statements of values, principles and objectives for improving the mental health of the population – and plans – more detailed sets of actions that allow for the implementation of policies by articulating activities, resources and time frames (World Health Organization, 2004). However, for this study *mental health policy document* refers to all mental health policies, strategies and plans.

Web searches were conducted in February 2014 to identify the most recent, publicly available, English language, online mental health policy documents from Australia, New Zealand, the United Kingdom (UK), Ireland and Canada. For Australia, the UK and Canada documents were sought nationally and from each jurisdiction (state, territory, province or country); New Zealand and Ireland do not have equivalent state or province levels of government. Therefore 29 jurisdictions were included in the search (including four UK countries, eight Australian states/territories and 13 Canadian provinces/territories). The terms ‘mental health policy’, ‘mental health strategy’ and ‘mental health plan’ were entered into the search engine Google along with the relevant jurisdiction, or into each jurisdiction's website (typically that of the Department of Health or its equivalent).

Identified policy documents were included in the review if they were officially endorsed by the jurisdiction and were the current or most recently available version for that jurisdiction, including possibly lapsed documents where no updated version could be identified. Policy documents focusing on one area of mental health, such as mental health promotion or psychosocial support services, were included; those focusing primarily on a sub-population (e.g. children or minority groups), suicide prevention, or drug and alcohol services were excluded. Mental health action plans covering a short timeframe of 1 year or less were also excluded because they may not represent a jurisdiction's whole policy.

### Data extraction

#### Objectives and guiding principles

Sections labelled as ‘objectives’ and ‘guiding principles’ were extracted from each policy document. In some cases, policies labelled these statements ‘strategic directions’, ‘goals’ or ‘priorities’; these were included if they expressed broad aims, underlying philosophies or areas to focus reform activities.

#### Relationships with other sectors

For each policy document, we recorded the sectors targeted for intersectoral collaboration and mechanisms by which collaboration was to be achieved. For each sector, the available detail was categorised as Level 1 where it was acknowledged that collaboration was important but no further information was provided, or Level 2 where details about the nature and structure of the collaboration were provided. The latter ranged from very specific intersectoral collaborations formalised in legislation to less far-reaching examples, such as pilot projects or mental health training in other sectors.

#### Examples of collaboration

A cross-section of the most detailed or comprehensive intersectoral policy initiatives covering the range of sectors identified was selected for further description. Details extracted for each example included the jurisdiction, policy document, sectors involved, programme description, linkage mechanisms and evidence of evaluation. For comparison across initiatives, mechanisms were classified using the taxonomy developed by Whiteford *et al*. (2014), which grouped strategies described in studies of intersectoral linkages between the mental health and non-health sectors into nine categories: joint service planning, a single multiagency care plan, formal interagency collaborative agreements, staff training, sharing of information systems, blended funding initiatives, joint service provision, service co-location and administration by a single lead agency.

### Information synthesis and analysis

One of the four authors (S. D., G. C., N. R. and B. B.) extracted information from each identified mental health policy document into a template (fields included source, scope, objectives, guiding principles, relationship with other sectors). The templates were reviewed by one author (G. C.) in order to identify areas of commonality and difference across policy documents. First, we looked for common themes in objectives/guiding principles and sectors that were identified by a number of jurisdictions. We then recorded whether these sectors or themes were present in each policy document; a second author (S. D. or C. M.) reviewed these ratings with reference to the original policy documents.

## Results

### Overview of policy documents

Thirty-eight current or recent mental health policy documents were identified, originating from 22 of the 29 eligible jurisdictions in the countries considered, including 16 national-level and 22 state- or province-level documents (Table 1). Policy documents for Northern Territory (Australia); Nunavut, Prince Edward Island, Saskatchewan and Yukon (Canada); Northern Ireland (UK); and an English language version for Quebec (Canada) could not be identified. Of the 38 included documents, three appeared to have lapsed but more recent documents could not be identified online (Department of Health and Human Services, 2006; NSW Department of Health, 2008; Queensland Health, 2009). Jurisdictions varied in the mix of documents available; the Australian national level, New South Wales and England all had a primary policy document supplemented by one specifically focusing on interagency collaboration.

**Table 1.** Objectives and guiding principles described in mental health policy documents

Jurisdiction^a^	Mental health policy document	Themes in objectives/guiding principles		
		Whole of government approach	Coordination and integration of services	Increased social and economic participation
Australia (national)	National Mental Health Policy 2008 (Commonwealth of Australia, 2009)	+	+	+
	Fourth National Mental Health Plan 2009–2014 (Australian Health Ministers Conference, 2009)	+	+	+
	The Roadmap for Mental Health Reform 2012–2022 (Council of Australian Governments, 2012)			+
Australian Capital Territory	ACT Mental Health Services Plan 2009–2014 (ACT Government, 2009)	+	+	+
	Building a Strong Foundation: Framework for Promoting Mental Health and Wellbeing in the ACT 2009–2014 (ACT Health, 2009)	+	+	
New South Wales	NSW: A New Direction for Mental Health 2006 (NSW Department of Health, 2006)		+	+
	Community Mental Health Strategy 2007–2012 (NSW Department of Health, 2008)	+	+	+
	New South Wales Interagency Plan for Better Mental Health 2008 (New South Wales Government, 2008)	+	+	
Queensland	Queensland Plan for Mental Health 2007–2017 (Queensland Health, 2008)	+	+	+
	Strategic Directions for Mental Health Promotion 2009–2012 (Queensland Health, 2009)		+	
South Australia	South Australia's Mental Health and Wellbeing Policy (SA Health, 2010)	+	+	+
Tasmania	Mental Health Services Strategic Plan 2006–2011 (Department of Health and Human Services, 2006)		+	
	Building the Foundations for Mental Health and Wellbeing (Department of Health and Human Services, 2009)	+	+	+
Victoria	Because Mental Health Matters, Mental Health Reform Strategy 2009–2019 (Mental Health and Drugs Division, 2009)	+	+	+
Western Australia	Mental Health 2020: Making It Personal and Everybody's Business (Government of Western Australia and Mental Health Commission, 2010)	+	+	+
	A Recovery Vision for Rehabilitation: Psychiatric Rehabilitation Policy and Strategic Framework (Office of Mental Health, 2004)			+
Canada (national)	Changing Directions, Changing Lives: The Mental Health Strategy for Canada (Mental Health Commission of Canada, 2012)	+		+
Alberta	Creating Connections: Alberta's Addiction and Mental Health Strategy (Government of Alberta, 2011*b*)		+	
	Creating Connections: Alberta's Addiction and Mental Health Action Plan 2011–2016 (Government of Alberta, 2011*a*)		+	
British Columbia	Healthy Minds, Healthy People: A Ten-Year Plan to Address Mental Health and Substance Use in British Columbia (Government of British Columbia, 2010)		+	+
Manitoba	Rising to the Challenge: A Strategic Plan for the Mental Health and Well-Being of Manitobans (Government of Manitoba, 2011)	+	+	+
New Brunswick	The Action Plan for Mental Health in New Brunswick (Government of New Brunswick, 2011)	+		+
Newfoundland and Labrador	Working Together for Mental Health: A Provincial Policy Framework for Mental Health and Addictions Services in Newfoundland and Labrador (Government of Newfoundland and Labrador, 2005)	+	+	+
Northwest Territories	Pathways to Wellness: An Updated Action Plan for Addictions and Mental Health 2014–2016 (Northwest Territories Health and Social Services, 2012)	+	+	
Nova Scotia	Together We Can: The Plan to Improve Mental Health and Addictions Care for Nova Scotians (Government of Nova Scotia, 2013)			
Ontario	Open Minds, Healthy Minds Ontario's Comprehensive Mental Health and Addictions Strategy (Ontario, 2011)		+	+
England	No Health Without Mental Health: A Cross-Government Outcomes Strategy for People of All Ages (HMG/Department of Health, 2011*a*)			+
	No Health Without Mental Health: Delivering Better Mental Health Outcomes for People of All Ages (HMG/Department of Health, 2011*b*)			+
	No Health Without Mental Health: Implementation Framework (Department of Health, 2012)			+
	Closing the Gap: Priorities for Essential Change in Mental Health (Department of Health, 2014)	+	+	
New Zealand	Te Tāhuhu: Improving Mental Health 2005–2015: The Second New Zealand Mental Health and Addiction Plan (Ministry of Health, 2006)	+		
	Rising to the Challenge: The Mental Health and Addiction Service Development Plan 2012–2017 (Ministry of Health, 2012)	+	+	
	Blueprint II. Improving Mental Health and Wellbeing for All New Zealanders – How Things Need to Be 2012 (Mental Health Commission, 2012*a*)			
	Blueprint II. Improving Mental Health and Wellbeing for All New Zealanders – Making Change Happen (a companion document to Blueprint II) 2012 (Mental Health Commission, 2012*b*)			
Republic of Ireland	A Vision for Change: Report of the Expert Group on Mental Health Policy (Government of Ireland, 2006)		+	+
Scotland	Mental Health Strategy for Scotland: 2012–2015 (The Scottish Government, 2012)		+	
Wales	Together for Mental Health: A Strategy for Mental Health and Wellbeing in Wales 2012 (Welsh Government, 2012*b*)	+	+	
	Together for Mental Health: Delivery Plan 2012–2016 (Welsh Government, 2012*a*)	+	+	
Total: jurisdictions (*n* = 22)		**16**	**19**	**16**
Total: countries (*n* = 5)		**4**	**5**	**4**

a Mental health policy documents were not able to be identified for the Northern Territory (Australia); Nunavut, Prince Edward Island, Saskatchewan and Yukon (Canada), and Northern Ireland (UK). An English language mental health policy document for Quebec (Canada) could not be located.

### Objectives and guiding principles

Recurring themes related to intersectoral policy identified in objectives/guiding principles were: a whole of government approach; coordination and integration of services; and increased social and economic participation. At least one of these themes was present in all but one jurisdiction's policies (Table 1).

#### Whole of government approach

The whole of government approach refers to involvement of multiple levels of government and multiple government agencies in mental health. This was an objective or guiding principle for three-quarters of jurisdictions, described in varying ways. For example, New South Wales’ Interagency Plan operationalised whole of government accountability and partnership by mapping all actions in three key areas – prevention and early intervention, community support and emergency responses – to responsible government departments. In contrast, many policy documents, such as those from Wales, expressed cross-government commitment to all sectors working together.

#### Coordination and integration of services

Nineteen of the 22 jurisdictions’ policy documents identified coordination or integration of services as an objective. In many documents, this was linked to promoting recovery or streamlining and improving service access for people with complex needs. For example, New Zealand's policy documents outlined a stepped care model, including investment in programmes such as housing and employment that actively facilitate return to natural community supports.

#### Increased social and economic participation

Three-quarters of jurisdictions included objectives linked to increasing social and economic participation. Policy documents described activities related to improving social inclusion, such as stigma reduction, which can be delivered within mental health, but also many which were the primary responsibility of non-health sectors. For example, the Canadian strategy identified full participation in work, education and community life through provision of the right services and supports as key to recovery for people with mental illness, stressing the role that schools, workplaces and other community settings play in full social and economic participation.

### Relationships with other sectors

**Fig. 1.**
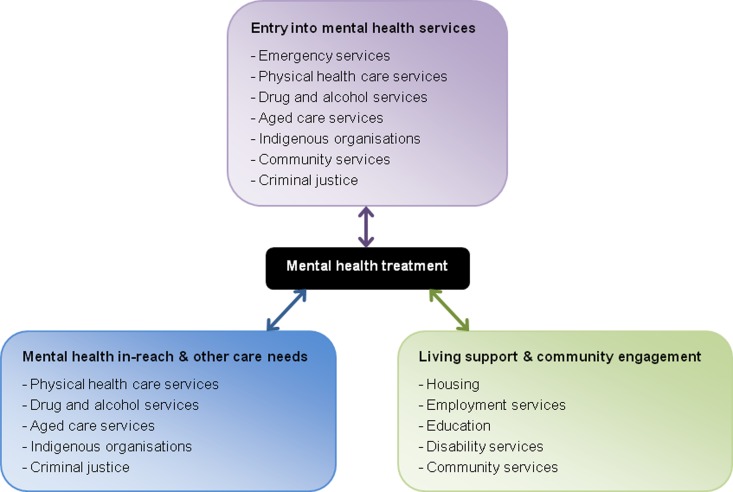
Key relationships between mental health and other sectors.

All jurisdictions’ policy documents acknowledged the need for collaboration with sectors other than mental health; however, some between-jurisdiction variations were evident. Key interfaces between sectors are shown in Fig. 1. Within health, sectors for collaboration identified by nearly all jurisdictions were drug and alcohol and physical health care services, while aged care was highlighted by all but one jurisdiction outside of Canada (Table 2). Non-health sectors identified as significant for collaboration by all or nearly all jurisdictions included employment services, education, housing, community services, criminal justice and Indigenous organisations where relevant. Disability services were recognised as important by two-thirds of jurisdictions, while half also identified emergency services (e.g. police and ambulance), the latter mainly in Australia.

**Table 2.** Relationship between mental health and other sectors described in mental health policy documents

Jurisdiction	Mental health policy document (abbreviated title)^a^	Relationship with other sectors										
		Physical health	Drug and alcohol	Aged care	Indigenous	Housing	Education	Employment	Disability	Community^b^	Emergency	Criminal Justice
Australia (national)	National Mental Health Policy	+	+	+	+	+	+	+	+	+		+
	Fourth National Mental Health Plan	++	++	++	+	++	++	+	+	++	++	++
	The Roadmap for Mental Health Reform	++	++		++	++	++	++	++	++	++	++
Australian Capital Territory	ACT Mental Health Services Plan	++	++	++	++	++	++	++		++		++
	Building a Strong Foundation	++					++	++				
New South Wales	NSW: A New Direction for Mental Health	++	++	++		++	++	++		++	+	++
	Community Mental Health Strategy	++	++		++	++	++	++	+	++	++	+
	Interagency Plan for Better Mental Health	++	++	++	++	++	++	++	++	++	++	++
Queensland	Queensland Plan for Mental Health	++	++	++	++	++	+	+	++	++	++	+
	Strategic Directions for Mental Health Promotion	++		+	+		++	+		+		
South Australia	Mental Health and Wellbeing Policy	+	+	+	+	+	+	+	+	+	+	+
Tasmania	Mental Health Services Strategic Plan				+	+		+		+		+
	Building the Foundations	++	++	+	++	++	++		++	++	++	++
Victoria	Because Mental Health Matters	++	++	++	++	++	++	++	+	++	++	++
Western Australia	Mental Health 2020	+	+	+	++	++	+	+	+	+	++	++
	A Recovery Vision for Rehabilitation	++		+	+		++	++		+		
Canada (national)	Changing Directions, Changing Lives	+	+		+	++	++	++	+	++		++
Alberta	Creating Connections Strategy	+	+		++	++	+	+		+		++
	Creating Connections Action Plan	+	++		++	++	++	+		++		+
British Columbia	Healthy Minds, Healthy People	+	++		+	++	+	++	++	++		++
Manitoba	Rising to the Challenge	+	++		+	++		+	+		+	+
New Brunswick	The Action Plan for Mental Health		+		++	++	++	++	+	++		++
Newfoundland and Labrador	Working Together for Mental Health	+	+		+	+	+	+		+		
Northwest Territories	Pathways to Wellness				++	+	++	+		+		++
Nova Scotia	Together We Can	+	+	+	+	++	++	+		+	++	++
Ontario	Open Minds, Healthy Minds	++	+			++	++	+		+	++	++
England	No Health Without Mental Health (Strategy)	++	+			++	++	++		+		++
	Delivering Better Mental Health Outcomes	++	+			++	++	++		++		++
	Implementation Framework	++	+	++		++	++	++	++	++		++
	Closing the Gap	++				++	++	++		+	++	++
New Zealand	Te Tāhuhu	+	+	+	+	+	+	+	+	+		+
	Rising to the Challenge	++	++	++	++	++	++	++	++	++		++
	Blueprint II	++	++	++	++	++	++	++	++	++		++
	Blueprint II (companion)	++	++	++	++	++	++	++	++	++	+	++
Republic of Ireland	A Vision for Change	++	++	++		++	++	++	++	++		++
Scotland	Mental Health Strategy for Scotland	++	++				+	++		++		++
Wales	Together for Mental Health: Strategy	++	++	++		++	++	++	++	++		++
	Together for Mental Health: Delivery Plan	++	++	++		++	++	++		++		++
Total: jurisdictions (*n* = 22)		**20**	**21**	**13**	**17**	**21**	**21**	**22**	**15**	**21**	**12**	**21**
Total: countries (*n* = 5)		**5**	**5**	**5**	**3**	**5**	**5**	**5**	**5**	**5**	**4**	**5**

a For full policy document titles and references see Table 1.

b May include some overlap with other sector categories, in particular disability and employment services.

+Level 1 relationship – intersectoral collaboration is acknowledged as important without further detail being provided.

++Level 2 relationship – further information about collaboration was provided, ranging from some explanation of an example of intersectoral collaboration with minimal details provided to highly detailed and specific examples.

#### Drug and alcohol services

Mental health and drug and alcohol services were described as an increasingly combined sector in some jurisdictions (e.g. New Zealand and Canada), whereas in others (e.g. many Australian jurisdictions), currently they were largely separate in their financing and governance. Policy documents from almost all jurisdictions acknowledged the need for these areas to work closely together to best cater for people with both mental health and drug and alcohol problems. Mechanisms for collaboration included establishing linkages between services, for example through co-location; improving communication and information sharing, such as through introducing shared systems of unique identifiers and access to consultation; and developing joint care plans.

#### Physical health care services

Most jurisdictions acknowledged the importance of collaboration between mental health and the broader health sector. Policy documents mainly focused on collaboration with primary care and acute health services, particularly emergency departments. Partnerships between mental and physical health services were identified as significant in facilitating entry into the mental health system and for maintaining good physical health in people with SPMI. Consultation-liaison and shared care arrangements between mental health and primary care were advocated to deliver comprehensive physical and mental health care.

#### Aged care services

Linkages between mental health and aged care featured frequently in policy documents from non-Canadian jurisdictions. These identified the importance of mental health providers working with aged care services to support older people with mental illness in their home or residential facility. The importance of good discharge planning involving all parties was also emphasised.

#### Employment services

Policy documents from all jurisdictions recognised the bidirectional relationship between employment and mental health, with employment services highlighted as a key area for collaboration. The main focus was on models which support people with mental illness to prepare for work, return to employment and remain employed. These rely on partnerships between clinical services, community support agencies, vocational training institutions, employment agencies and businesses, and include models such as Individual Placement and Support.

#### Education

Collaboration with the education sector was emphasised as particularly important for mental health promotion, and prevention of and early intervention for mental illness, but also for maintaining engagement with education for young people with severe mental disorders. Some policy documents focused on governance arrangements, proposing shared local and regional service agreements between, for example, schools, community-based mental health services and child protection services. Others focused on service delivery, ensuring that school teaching and counselling staff access relevant training and support from mental health specialists.

#### Housing

All but one jurisdiction highlighted the need to develop better partnerships between government departments responsible for mental health, housing and other areas such as disability services. Policies highlighted difficulties faced by people with SPMI in accessing and maintaining housing, especially during repeated or prolonged inpatient stays, and the interaction between homelessness and poor mental health.

#### Community services

Nearly all jurisdictions identified the importance of linking with community services to facilitate recovery. This category represents a broad group of services, including women's, transcultural, sexual assault, child and youth, and family support services. As a broad descriptor, community services may also have been used in policy documents to include services considered under other sections, such as disability and employment services.

#### Criminal justice

The need for mental health to collaborate with the justice sector was acknowledged in policy documents from all but one jurisdiction. This reflects concern that prisoners and ex-prisoners have high rates of mental illness and often other complex needs. Justice systems, including youth justice services, were identified as providing a unique opportunity to screen, identify, and connect or reconnect with people with untreated mental illness through police and court diversion.

#### Indigenous organisations

Relationships between mental health and Indigenous organisations were given prominence in many policy documents. Partnerships with services for Indigenous populations in Australia (Aboriginal and Torres Strait Islander), Canada (First Nation, Métis and Inuit) and New Zealand (Māori) were identified as essential for delivering culturally appropriate and holistic health services. Collaborations between mental health, Indigenous primary health organisations, ministries for Indigenous affairs and Indigenous community leaders were emphasised.

#### Disability services

Collaboration between mental health and disability services was identified in policy documents from approximately two-thirds of jurisdictions, and recognised as influential for recovery. This includes access to general community support provided for people with a range of disabilities (e.g. physical, intellectual and mental), as well as support services tailored specifically for people with disabling mental disorders.

#### Emergency services

The need for collaboration with emergency services (e.g. ambulance and police) was flagged in policies from half of jurisdictions, the majority in Australia. Emergency services are sometimes involved in instigating mental health care, such as where a person is deemed to be a risk to themselves or others. Jurisdictions noted the need for better coordination between mental health crisis teams, ambulance services and police. Identified mechanisms for collaboration included provision of mental health training to frontline emergency workers, collaborative development of protocols guiding transitions between sectors, and rapid access to specialised services for emergency staff.

### Examples of collaboration

A wide range of intersectoral policy initiatives were described in varying depths. Some more detailed or comprehensive examples are profiled in Table 3. These examples are broadly representative of the range of initiatives covered but not exhaustive. Policy documents from New South Wales, New Zealand, Wales and England had a particularly strong focus on intersectoral approaches and therefore provided a greater number of detailed examples.

**Table 3.** Examples of intersectoral collaboration efforts described in mental health policy documents

Jurisdiction	Other sectors involved	Initiative	Linkage mechanisms^a^								
			1	2	3	4	5	6	7	8	9
Ireland (Government of Ireland, 2006)	Physical health	Referral protocols, integrated care pathways and shared care arrangements (including consultation liaison). Mental health training for GPs and other primary care providers	+		+	+			+		
Ontario (Ontario, 2011)	Physical health, Drug and alcohol	Better coordination with addictions, family health, acute care and emergency departments through quality improvement (e.g. collaborative services), automated functions (e.g. common intake and referral; collaborative service plans), accountability agreements and standards	+	+	+						
Australian Capital Territory (ACT Government, 2009)	Drug and alcohol	Cooperative partnership involving consultation and supervision, reciprocal placements, training, and strong leadership. Mental health comorbidity clinician located part time in drug and alcohol services and vice versa. Commitment to develop an Integrated Comorbidity Strategy	+		+	+				+	
England (Department of Health, 2012)	Aged care	*Doncaster Care Home Liaison Service* provides rapid access to mental health services for older people in registered care homes. Delivery of educational packages and advice to care home staff	+			+					
Victoria (Mental Health and Drugs Division, 2009)	Indigenous	Collaborative arrangements between Aboriginal Health Services, Aboriginal Community Controlled Health Organisations, local Aboriginal organisations and mental health services to provide culturally supportive social and emotional wellbeing and recovery services	+								
Northwest Territories (Northwest Territories Health and Social Services, 2012)	Indigenous	Collaboration between mental health and Aboriginal and community governments to develop action plans building on existing community assets. Joint provision of on-the-land and traditional healing options for mental health and addictions	+		+				+		
New South Wales (New South Wales Government, 2008)	Housing, Disability	*Housing and Support Initiative (HASI)*: three-way partnership – tenancy management provided by housing, support services by disability organisations, and clinical care by mental health. *Joint Guarantee of Service* outlining roles and responsibilities of participating agencies	+		+						
New Brunswick (Government of New Brunswick, 2011)	Housing, Employment	Deputy ministerial committee representing all relevant government departments to provide oversight in implementing the mental health plan. Common consent form for disclosure of personal information and inter-departmental case management to ensure continuity of service	+				+		+		
Western Australia (Office of Mental Health, 2004)	Education	Education assistants employed by the Department of Education and Training to support students with mental illness to remain in mainstream education. Applications are considered by a multidisciplinary committee	+								
Ontario (Ontario, 2011)	Education	Collaboration with schools to provide mental health programmes – mental health workers and nurses in schools provide direct care and connect with mental health services. Mental health literacy training for educators	+			+				+	
British Columbia (Government of British Columbia, 2010)	Employment	Collaboration with employers and unions to develop and implement workplace supports (e.g. self-care resources), early identification of problems, and links to services for individuals re-entering work and moving towards recovery	+			+					
England (HMG/Department of Health, 2011*a*)	Employment	Employment coordinators in every area who work in conjunction with local Jobcentre Plus offices, employers and occupational health schemes to help people with mental illness enter, stay in and return to employment	+							+	
Canada (Mental Health Commission of Canada, 2012)	Community	*Journey to Promote Mental Health:* collaboration between mental health and immigration providing training for front-line workers from community settlement agencies to raise awareness of mental health issues faced by newcomers, and enhance capacity to provide support and intervention				+					
New Zealand (Ministry of Health, 2012)	Community	Partnership with Child, Youth and Family department to coordinate service delivery, identify and address mental health needs for children in care, and provide services tailored to youth with complex needs and their families	+								
New South Wales (New South Wales Government, 2008)	Emergency	Formal memorandum of understanding for agencies involved in mental health emergency response, including a patient journey flowchart setting out agency roles at each stage. Formal interagency coordination committee and network of Interagency Local Protocol Committees	+		+						
England (Department of Health, 2014)	Emergency	*Street triage:* mental health professionals work with police as a first-line response – on the street or through a dedicated phone line. For non-criminal disturbances, police can call in mental health staff for rapid needs assessment and to direct the individual to appropriate help	+						+		
Western Australia (Government of Western Australia and Mental Health Commission, 2010)	Criminal justice, Emergency	Transport for people with mental illness to and from hospital and court that does not involve uniformed police and police vehicles. Working with police to divert people with mental health problems from being charged. A safe and secure facility for detention and treatment of accused offenders who are unable to stand trial because of unsoundness of mind	+								
Wales (Welsh Government, 2012*b*)	Criminal justice, Community	Collaboration between police, health and social services in use of places of safety. *Criminal Justice Liaison Services* in police custody and courts to facilitate access to treatment. Mental health advice and reports to custody suites and courts. Timely transfer of prisoners to hospitals under the Mental Health Act. Multi-disciplinary risk assessment, case management, support and rehabilitation prior to and at release from prison	+				+		+		

a Intersectoral linkage mechanisms (Whiteford *et al*. 2014), as detailed in policy documents:

1. Joint service planning and information exchange with interagency coordinating committees and/or intersectoral/interface workers.

2. A single multiagency care plan for each client.

3. Formal interagency collaborative agreements or memoranda of understanding.

4. Staff training, including joint training – ensuring staff have shared attitudes and consistent understanding.

5. Information-sharing using a single information system, shared case records or client tracking systems.

6. Blended funding initiatives.

7. Joint service provision through multidisciplinary, multiagency teams coordinated via regular communication.

8. Service co-location.

9. Service administration by a single lead agency.

Across jurisdictions, the sectors for which interagency initiatives were most frequently elaborated were housing, education, employment services and criminal justice. The most commonly identified mechanism for collaboration was joint service planning, often through interagency coordinating committees (Table 3). A number of jurisdictions (e.g. England, Ireland and Manitoba) also described established cross-government committees for social inclusion or public health which would address mental health as part of their broader mission. Other common linkage mechanisms identified across sectors included intersectoral liaison workers, such as mental health employment coordinators or mental health staff working into drug and alcohol services and schools; formal interagency agreements; joint training or cross-training of mental health and other staff to ensure shared understanding; and joint service provision through multidisciplinary, multi-agency teams. There was little discussion of financing arrangements for specific intersectoral initiatives in policy documents, with none of the examples in Table 3 clearly mentioning blended funding initiatives (e.g. sectors pooling funding for integrated services). However, it is likely that many standalone initiatives have dedicated funding; we did not specifically analyse financing arrangements.

Most examples of intersectoral collaboration in policy documents did not reference evidence for specific initiatives. Exceptions in Table 3 include: England's Doncaster Care Home Liaison Service, which cited evidence that the service led to reduced use of antipsychotics, mental health admissions and readmission rates; New South Wales's Housing and Support Initiative, shown to reduce the number and length of hospital admissions, improve frequency and quality of contact with family, and decrease substance abuse problems; and Canada's Journey to Promote Mental Health programme, found to improve community workers’ ability to identify mental illness and to reduce stigma. The Individual Placement and Support model for employment services was identified by several jurisdictions with reference to its strong evidence base for increasing competitive employment rates.

## Discussion

Our review of mental health policy documents from Australia, New Zealand, the UK, Ireland and Canada revealed remarkable consistency in their emphasis on whole of government and integrated, cross-sector approaches to influence recovery for people with mental illness. These similarities suggest that jurisdictions drew on comparable evidence and learned from strategies adopted in other countries. Sectors consistently identified as important across jurisdictions mirror the areas of social disadvantage experienced by people with SPMI, such as physical health care, employment and housing. Linkages between mental health and some sectors, in particular emergency, disability and aged care services, were emphasised more in certain jurisdictions, which may reflect differences in health and social services arrangements. There was wide variation in the level of detail on intersectoral programmes provided in policy documents. Nonetheless the evidence collected demonstrates that a variety of strategies, often multi-pronged approaches, are being trialled to facilitate intersectoral collaboration.

### Limitations

Our search method identified mental health policy documents in the public domain. We did not approach jurisdictions directly to obtain any additional policies, so it is possible that some documents were missed. Further, at least three of the included policy documents were out of date. It is unclear if these documents reflect the current status of mental health policy and services in the relevant jurisdictions, as we were unable to identify newer versions. The possible exclusion of a small number of policy documents or jurisdictions is unlikely to have impacted findings significantly, since there was consistency in policy directions across included documents and jurisdictions. This review was limited to intersectoral initiatives described in mental health policy documents, but it would also be informative to review intersectoral actions relevant to mental health endorsed in policy documents from other sectors, such as housing and social services.

The analysis of objectives and guiding principles identified prominent themes which were commonly highlighted by jurisdictions as areas of focus. However, jurisdictions not emphasising the common themes in these sections may have noted the importance of intersectoral collaboration within other parts of their policy documents. Objectives or sectors for collaboration were omitted from the overarching analysis if they were identified by only one or very few jurisdictions.

The study relied on information contained in overarching mental health policy documents. Jurisdictions may have well-developed collaboration efforts which were not detailed in these documents. For example, Battams & Baum (2010) found that collaboration between mental health and housing in South Australia was often more developed at local service levels than at the policy or state planning level. Although local grass-roots efforts at collaboration across sectors may exist, large-scale programmes and high level cross-government actions are unlikely to be implemented without an overarching policy direction. Alternatively, little detail in policy documents may indicate lack of a clear plan to implement collaboration beyond a strategic vision. The reviewed policy documents described the state of current services, jurisdictions’ objectives and plans, and programmes currently under development or in pilot phases; this information was usually several years old. It is difficult to judge how many of these plans have actually been implemented and with what success; therefore we focused on examples of actions rather than future plans.

### Implications

Intersectoral collaboration efforts should be evidence informed. The sectors for collaboration identified in policy documents were largely aligned with those identified as important for mental health outcomes in the literature (e.g. Lee *et al*. 2013; Cashin, 2014; Whiteford *et al*. 2014). The linkage mechanisms most frequently promoted, such as joint service planning, formal interagency agreements and staff training, also appear to be consistent with previous reviews and likely appropriate for facilitating intersectoral linkages (Whiteford *et al*. 2014). However, the rationale and/or evidence base used by jurisdictions to justify specific initiatives was generally not described in policy documents, though may be provided elsewhere.

Recognition of the need for intersectoral initiatives and plans to improve collaboration are important steps, but these plans need to be implemented, monitored and refined in order to improve outcomes. WHO (2004) outlines five steps to mental health policy advancement, including development, implementation, monitoring, evaluation and reformulation. Successful implementation requires appropriate funding models and adequate resources such as staff, funding and organisational support; however, details about these provisions were generally lacking in policy documents. This is noteworthy, given that Whiteford *et al*. (2014) identified constraints in resources such as funding, time, workloads and technology as the most commonly described barriers to intersectoral linkages. By way of example, blended funding initiatives were not described in any of our detailed collaboration examples, although they were a linkage mechanism used in one-quarter of studies (albeit mostly from the USA) reviewed by Whiteford *et al*. (2014). Resourcing should be part of the planning process. Programmes also need to be evaluated after implementation to ensure they are effective, particularly in improving outcomes for people with SPMI. There was generally little or no information provided about accountability and monitoring of collaboration efforts. Future intersectoral policy development would benefit from the inclusion of targets for ongoing monitoring and evaluation of outcomes. Evaluations will inform not only policy development in the relevant jurisdiction but also the adaptation of successful initiatives to other countries.

As mental health service systems evolve, it is helpful to share strategies and experiences from both high- and low- or middle-income countries (World Health Organization, 2004). Governments should consider the range of mechanisms and programmes through which coordination across sectors can be achieved. The most comprehensive examples described in mental health policy documents from the reviewed countries generally utilised multiple linkage strategies, with a particular focus on joint service planning through interagency coordinating committees. The policy approaches and examples of intersectoral collaboration profiled in this paper may provide a useful resource for jurisdictions endeavouring to expand coordination across sectors for mental health. These examples are a starting point for the gathering of evidence for effective initiatives and for tailoring these strategies to local contexts.

In low- and middle-income countries, like high-income countries, the focus of recent mental health reform has been on scaling up services in the community and integration with general health care (Eaton *et al*. 2011). Coordination across sectors for people with SPMI is just as critical in these settings. The Mental Health and Poverty Project reviewed mental health policies in Ghana, South Africa, Uganda and Zambia, finding broad recognition of the need for collaboration within health and to some extent with other sectors such as housing (Faydi *et al*. 2011). Similar to the current study, policies from these countries lacked detail on the specific roles and responsibilities of different sectors and the nature of collaboration. Further work in South Africa (Skeen *et al*. 2010) identified a similar list of sectors for collaboration to the current study (e.g. primary health care, substance abuse, the elderly, housing, education, employment, welfare and justice), and an overview of intersectoral responsibilities in removing barriers to service delivery for people with mental illness which was broadly comparable with the strategies profiled here.

Gaps and strategies for intersectoral coordination identified in this policy review are likely to be applicable to other countries, including lower resource settings. This review highlights the importance not only of endorsing a policy of intersectoral collaboration, but also clearly outlining responsibilities, resourcing, monitoring and evaluation processes for cross-sector initiatives. Countries with developing mental health systems may have a unique opportunity to focus on building integrated, person-centred systems of care across sectors which avoid some of the service fragmentation present in many more established systems.
